# Psychosocial Risk Factors of Quality of Life Outcomes Among Older Adults Living With HIV

**DOI:** 10.1093/geroni/igab046.1635

**Published:** 2021-12-17

**Authors:** Monique Brown

**Affiliations:** University of South Carolina, Columbia, South Carolina, United States

## Abstract

Antiretroviral therapy, higher education, and HIV disclosure have been linked to improved quality of life (QoL) among people living with HIV. However, research examining psychosocial risk factors of QoL among older adults living with HIV (OALH) is lacking. Therefore, the main aim of this study was to examine the psychosocial risk factors of QoL among OALH. Data were obtained from 156 adults aged 50 and older living with HIV in South Carolina. Multivariable regression models adjusting for sociodemographic characteristics were used to determine the association between psychosocial risk factors and QoL domains among OALH. Stigma was associated with the physical (β=0.058, p=0.023), social (β=-0.149, p=0.006), and spiritual (β=0.124, p=<0.001) domains. Resilience was associated with the psychological (β=0.206, p=<0.001), independence (β=0.100, p=0.010), social (β=0.166, p=0.004), and environmental (β=0.312 p=<0.001) domains. Depression and experiencing trauma were also associated with varying QoL domains. Findings may inform interventions geared towards improving QoL among OALH.

